# Slow light bimodal interferometry in one-dimensional photonic crystal waveguides

**DOI:** 10.1038/s41377-020-00460-y

**Published:** 2021-01-14

**Authors:** Luis Torrijos-Morán, Amadeu Griol, Jaime García-Rupérez

**Affiliations:** Nanophotonics Technology Center, Universitat Politècnica de València, 46022 Valencia, Spain

**Keywords:** Photonic crystals, Silicon photonics, Optical sensors, Slow light

## Abstract

Strongly influenced by the advances in the semiconductor industry, the miniaturization and integration of optical circuits into smaller devices has stimulated considerable research efforts in recent decades. Among other structures, integrated interferometers play a prominent role in the development of photonic devices for on-chip applications ranging from optical communication networks to point-of-care analysis instruments. However, it has been a long-standing challenge to design extremely short interferometer schemes, as long interaction lengths are typically required for a complete modulation transition. Several approaches, including novel materials or sophisticated configurations, have been proposed to overcome some of these size limitations but at the expense of increasing fabrication complexity and cost. Here, we demonstrate for the first time slow light bimodal interferometric behaviour in an integrated single-channel one-dimensional photonic crystal. The proposed structure supports two electromagnetic modes of the same polarization that exhibit a large group velocity difference. Specifically, an over 20-fold reduction in the higher-order-mode group velocity is experimentally shown on a straightforward all-dielectric bimodal structure, leading to a remarkable optical path reduction compared to other conventional interferometers. Moreover, we experimentally demonstrate the significant performance improvement provided by the proposed bimodal photonic crystal interferometer in the creation of an ultra-compact optical modulator and a highly sensitive photonic sensor.

## Introduction

The slowing down of light was first theoretically described by Hendrik Lorentz more than a century ago, when it was shown that the group velocity can be drastically decreased in the presence of an ultracold atomic vapour^1^. More recently, in 1999, these predictions were experimentally demonstrated, and a light speed of just 17 m/s was achieved using an electromagnetically induced transparency quantum phenomenon^2^. This intriguing finding captured the interest of the research community and gave rise to significant work aimed at producing slow light in solids at room temperature. Coherent population oscillation processes were introduced years later to solve this issue, and the feasibility of this effect was shown in ruby and alexandrite crystals^3,4^. Likewise, material engineered structures can create artificial optical resonances and produce slow light. This is the case for photonic crystal (PhC) structures that consist of periodic dielectric repetition for any of the three spatial dimensions and where the group velocity of the supported modes is dramatically reduced near the edge of the Brillouin zone^5^. PhCs can also exhibit one or several photonic bandgap (PBG) regions, in which certain frequencies are not allowed to propagate through the structure and where slow light is produced near the edge of the bands defining these PBGs^6^. Additionally, slow light behaviour can be observed for guided modes propagating through linear defects introduced in PhC structures. For instance, two-dimensional (2D) hole patterned PhC waveguides were exploited to show the active control of light, and an over 300-fold reduction in the group velocity was achieved on a compact silicon integrated circuit^7^. Slow light also enables us to temporarily store optical signals or provoke a stronger light–matter interaction that enhances optical phase non-linearities^8,9^, among other advantages. Nonetheless, PhCs present some limitations regarding the operating bandwidth as well as coupling losses and the tuning of the slow modes^10^.

In this context, three-dimensional (3D) and 2D PhCs have been used in subsequent years for the creation of 3D PBGs at near-infrared wavelengths^11^, ultra-compact optical switches^12^, refractive index (RI) biosensors^13^ or near-zero RI materials^14^. However, the combination of these types of PhCs with other integrated structures can be challenging due to their structural complexity, which hinders fabrication processes for mass production^15^. Instead, more straightforward designs based on one-dimensional (1D) periodic waveguides ease the fabrication process while preserving the slow light benefits of phase non-linearities and dispersion tunability^16^. Furthermore, these structures have a smaller lateral size in comparison with 2D PhCs, which reduces the footprint of the device. These were first demonstrated for the propagation of ultrashort pulses near the band edge with large group delays^17^ and as short resonators in planar integrated platforms^18^. Quasi-one-dimensional PhCs based on Bragg grating structures were subsequently employed for refractometric sensing^19^, and 1D PhC waveguides were reported for integrated tuneable time delay devices^20^ and negative group velocity anomalous phenomena^21^. These types of corrugated waveguides were demonstrated with low propagation losses below 1 dB^22^, which had a significant impact years later in the development of several applications, such as mid-infrared slow light engineering waveguides^23^ or label-free biosensors^24^. 1D PhC structures have also been extensively used in PhC enhancement microscopy applications, as in the case of enhanced fluorescence to quantify the concentration of a certain analyte in a liquid sample^25^.

Since the early 1990s, optical interferometry based on Mach-Zehnder interferometer (MZI)-integrated schemes has been extensively studied for the development of devices such as silicon modulators^26^ and biosensors^27^. Basically, in an MZI, light is split into two different optical paths and recombined to create an interference pattern at the output signal. Changes in the real part of the material RI induced, for example, by a temperature change or by an applied electric field produce a relative phase shift with respect to the reference arm. MZI-based systems have since been employed in making compact high-speed low-power consumption silicon modulators^28,29^, as well as high-performance integrated devices for biosensing^30^. Nevertheless, in all of the abovementioned approaches, the performance of the MZI scales with the length of the optical paths, which makes it very difficult to design compact interferometers with high operational features. Accordingly, plasmonic interferometers have been introduced in recent years to overcome some of these drawbacks, creating high-speed ultra-compact and low energy consumption modulators^31–33^. Similarly, nano-slits in a thin metal film have been employed to develop plasmonic MZI on-chip biosensors with very high bulk RI sensitivity^34^. Moreover, other MZI configurations involving novel materials such as graphene, indium tin oxide (ITO) and lithium niobate have been investigated for their broadband and efficient electro-optical responses^35–37^. Although these interferometers offer prominent breakthroughs in comparison with classic dielectric MZI configurations, the use of new materials makes the fabrication processes complex and adds extra costs and difficulties to the micro-structuring of photonic circuits.

Integrating all-dielectric slow light elements in MZI schemes offers some advantages in terms of footprint reduction for the final device while maintaining a fully silicon-based structure. This idea was initially introduced at the end of the last century, where grating structures were included in the optical paths of an MZI modulator to improve its efficiency by reducing the group velocity of the propagating modes^38^. Later, this concept was extended to 2D PhCs to develop a highly compact asymmetric MZI of only 20 μm in length^39^, as well as high-speed low-voltage modulators employing polymer-infiltrated materials^40^ or embedded PhC cavities based on electro-optic^41^ or thermo-optic effects^42^ with switching speed limitations^43^. For 1D PhC waveguides, high-speed electro-optic modulators of 500 μm length, including a corrugated waveguide in one of the MZI arms, have been proposed for a dense integration level in foreseeable network-on-chip devices^44^. Similar MZI designs, including highly dispersive 1D periodic structures made of embedded circular holes in rectangular waveguides, have also been validated for achieving very short biosensing devices^45^. However, classic MZI-based configurations require additional photonic structures, such as power splitters or different optical waveguides, to perform interferometry. In this scenario, bimodal waveguide sensors were proposed to address limitations regarding compactness and ease of fabrication^46^. The underlying concept of these interferometers relies on exciting the first two electromagnetic modes that have the same polarization in an optical waveguide and making them interfere, converting a change in RI into an intensity modulation. Since both modes do not interact equally with an induced RI change, a phase shift is therefore produced between the fundamental mode, acting as a reference, and a higher-order mode, acting as an active mode. The higher-order mode is more sensitive to changes in the RI than the fundamental mode in a conventional MZI sensing arm, leading to a higher accumulated phase shift. This principle has been extensively studied in recent work for biosensing applications in lab-on-a-chip platforms^47^. Other bimodal concepts have also been proposed by our group for ultra-high spectral-based sensitivity in silicon periodic structures in the subwavelength regime^48,49^. Nonetheless, in this latter case, the operation principle is different from that of standard bimodal waveguides since both modes present similar dispersion properties and equally interact with the surrounding medium and thus create very large shifts in the spectral interferences.

In this work, we propose a short and single-channel bimodal interferometer enabled by all-dielectric 1D PhC waveguides working in the slow light regime at telecom wavelengths. We optimize and experimentally demonstrate the periodic structure to support a dispersive higher-order mode (acting as an active mode) with a drastically reduced group velocity in comparison to the fundamental mode (acting as reference). Our design encompasses benefits from PhCs and bimodal-based interferometers in terms of sensitivity and compactness by including slow light elements of straightforward 1D designs. We also experimentally demonstrate the operation of these interferometers when temperature and cladding RI changes are produced, confirming them as promising alternatives for high-efficiency modulators and high-sensitivity RI sensors, respectively, both with extremely reduced footprints.

## Results

### Principle of operation

**Fig. 1:**
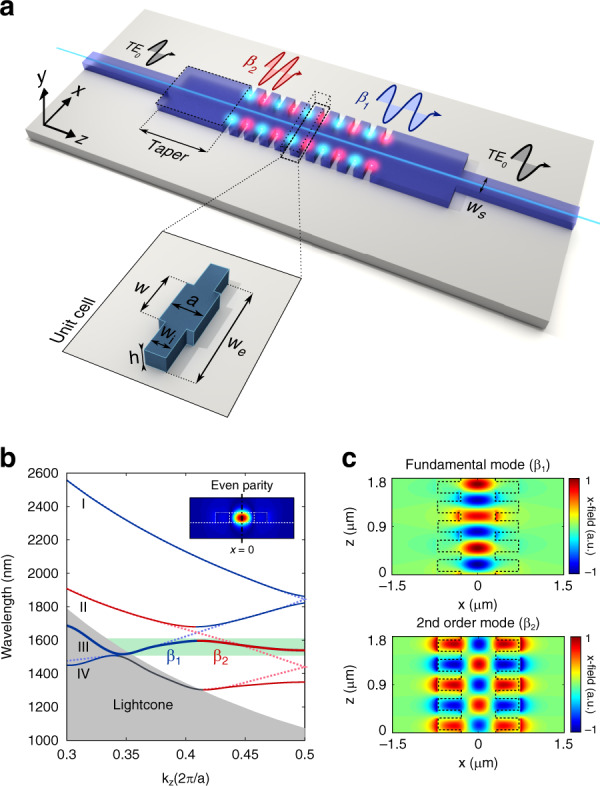
Design and simulation of the 1D PhC. **a** 3D sketch of the proposed design composed of two single-mode waveguides at the input/output ports and a rectangular taper between the uniform and periodic bimodal parts. The inset shows the unit cell dimensions of the 1D PhC. **b** Dispersion diagram of the 1D PhC showing the first three *x*-even parity bands for TE-like polarization. The blue lines depict the contribution of the fundamental mode to each band, and the red lines show the contribution of the higher-order mode. Dashed lines represent the hypothetical behaviour of the modes for a uniform non-periodic structure. The green shaded area indicates the bimodal region created in the proposed structure. The design parameters are *a* = 370 nm, transversal element width *w*_*i*_ = 220 nm, transversal element length *w*_e_ = 1400 nm, central waveguide width *w* = 600 nm and height *h* = 220 nm, and single-mode waveguide width *w*_s_ = 450 nm. **c** Real part of the electric field *x*-component for the fundamental and higher-order modes in the *xz* plane for *y* = 0. The field patterns are calculated in the third band for *k*_*z*_ = 0.3652*π*/*a* and *k*_*z*_ = 0.4752*π*/*a*. The black dashed line represents the geometric shape of the silicon 1D PhC structure

The proposed design, which is shown in Fig. 1a, is fully based on a silicon structure surrounded by silica cladding, in which a single-mode input waveguide, supporting the fundamental mode of transverse electric (TE) polarization, transfers its power to the first two TE-like even modes in the bimodal region of the 1D PhC: TE_0_-like and TE_2_-like. These two modes propagate through the 1D PhC and, after a certain distance, interfere in the abrupt discontinuity with the exit single-mode waveguide and thus contribute to the excitation of the fundamental TE mode at the output. Therefore, the transferred power may be expressed as a function of the phase shift accumulated between both modes in the bimodal region. Consequently, as occurs with a conventional MZI, by measuring the interference pattern in the transmission spectra, we obtain information about the phase shift between both modes and how it varies when a change in the RI is induced. In addition, a rectangular taper is placed in the transition between the single-mode waveguide and the bimodal PhC waveguide for efficient modal excitation in the periodic section. The bimodal interferometric structure is composed of a repetition of a basic unit cell consisting of a transversal element (or corrugation) over a central rectangular waveguide (see the inset of Fig. 1a), thereby creating a 1D PhC in the *z*-axis propagation direction. The design parameters of the proposed unit cell are a lattice period *a* = 370 nm, transversal element width *w*_*i*_ = 220 nm, transversal element length *w*_e_ = 1400 nm, central waveguide width *w* = 600 nm and height *h* = 220 nm, accessed in and out with a single-mode waveguide of width *w*_s_ = 450 nm.

Figure 1b depicts a dispersion diagram of the designed 1D PhC in the irreducible Brillouin zone, showing the TE-like bands that will be excited by the fundamental TE mode of the single-mode input waveguide. Only those bands presenting an even parity with respect to the *x* = 0 plane are considered, since the odd parity modes will not be excited, as in the case of the first-order mode (TE_1_-like), due to symmetry conditions in the interface between the single-mode waveguides and the bimodal waveguide. The first three bands are depicted and show the contributions of the fundamental (TE_0_-like) and second-order (TE_2_-like) modes in blue and red, respectively. The first band (I) is completely formed by the fundamental mode, while the second and third bands (II and III) are a combination of both modes. A PBG is created between these two bands as a result of the anti-crossing point produced by the fundamental mode, folded into the first Brillouin zone, and the higher-order mode. In PhC theory, when two modes of the same polarization and parity intersect, they couple, and the bands repel^50^, producing a dispersive behaviour similar to that obtained when a band reaches the edge of the irreducible Brillouin zone^51^ (*k*_*z*_ = 0.5 2*π*/*a*). Consequently, we obtain two different bimodal regimes near the PBG for the second and third bands, although in this work, we focus on the third band since lower group velocities can be achieved in this operating region. Therefore, in the third band (III), a bimodal behaviour with two different propagation constants and field patterns (see Fig. 1c) is obtained for wavelengths of ~1550 nm, which is the region of interest (ROI) for our purposes (shaded green area in Fig. 1b). The fundamental mode is strongly confined within the central waveguide of the PhC, while the higher-order mode is partially localized in the transversal elements of the structure. Consequently, the higher-order mode strongly interacts with the periodic pattern of the structure, thus producing a highly dispersive behaviour of the third band at the end of the Brillouin zone. The group velocity, which is mathematically described as the derivative of the angular frequency with respect to the wave vector (*v*_*g*_ = ∂*ω*/∂*k*), is given by the slope of the bands for each *k*_*z*_ value in the dispersion diagrams previously shown. Therefore, at high wavelengths in the ROI, slow light is produced for both modes, which is not suitable for our purposes. In contrast, at lower wavelengths, only the higher-order mode becomes slow light as it nears the edge of the Brillouin zone, while the fundamental mode shows normal dispersive behaviour, thus achieving a high group velocity difference between both modes in this wavelength region.

**Fig. 2:**
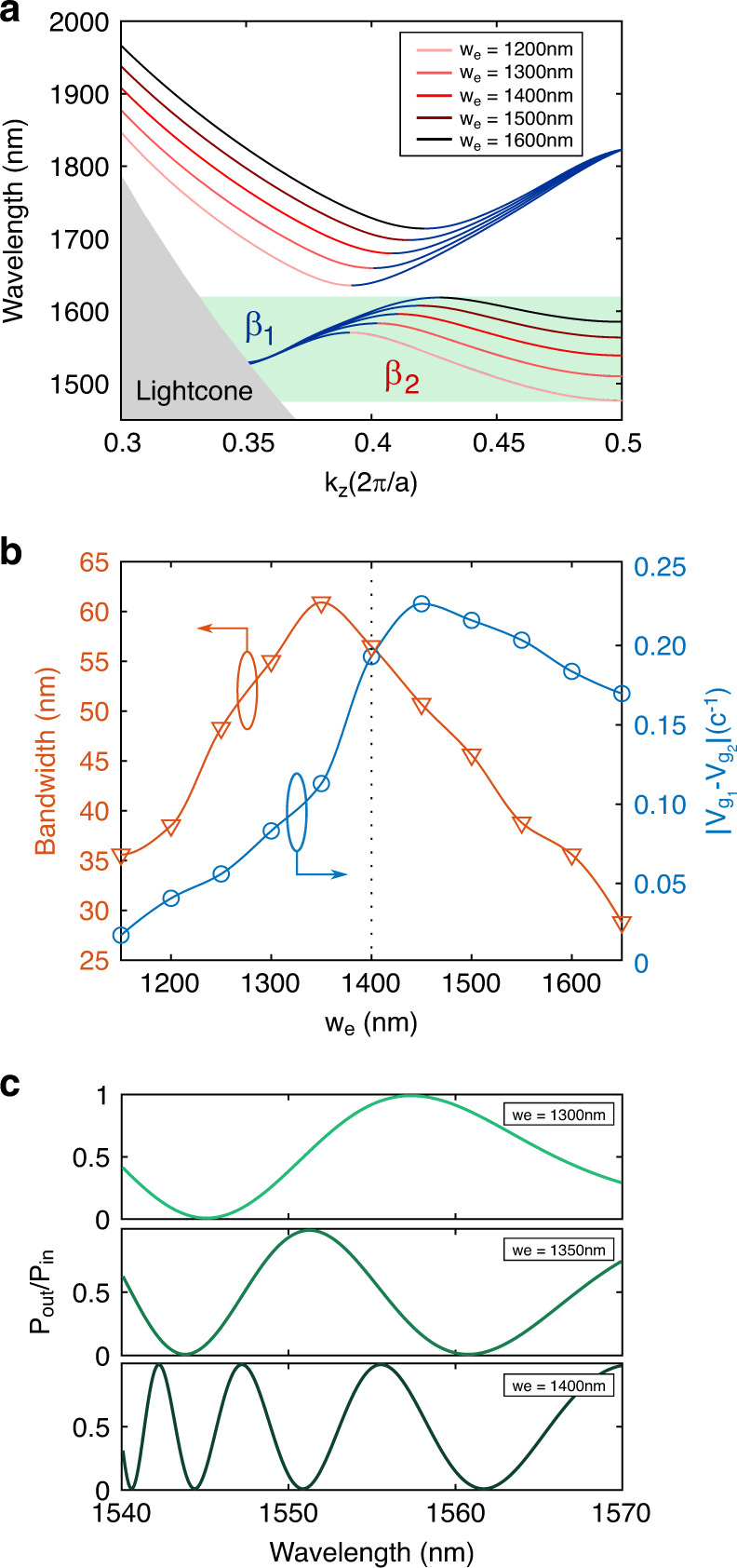
Optimization of the band structure. **a** Second and third bands for different transversal element lengths *w*_e_ (the rest of the design parameters are the same as previously detailed). The contributions of the fundamental and higher-order modes to band formation in the bimodal region are depicted in blue and red, respectively. **b** Trade-off between the group velocity difference between both modes (in blue) and the bimodal bandwidth (in orange) as a function of *w*_e_. The group velocity difference was calculated for those wavelengths in the lower edge of the bimodal ROI where a slow light behaviour is obtained for the higher order. **c** Ratio between the output and input power, calculated as the squared cosine of the phase shift as a function of the wavelength. Various *w*_e_ lengths are considered, representing the interference pattern evolution as a result of the slow light behaviour

Under induced RI variations in the system, the effective index of the higher-order mode will be drastically changed in comparison to the effective index of the fundamental mode, which acts as a reference. Accordingly, slowing the higher-order mode critically enhances the phase shift accumulated when a change in the RI is induced. Hence, an effect is obtained similar to what happens in an MZI when the arm length is increased to achieve higher phase shifts, but in this case, it drastically slows down the higher-order mode. To achieve this, we focus on studying the influence of the design parameters on the bimodal ROI in the third band. In Fig. 2a, we can observe the evolution of the dispersion relations for the second and third bands for different values of the parameter *w*_e_. The rest of the design dimensions are kept as detailed above to obtain a bimodal behaviour around 1550 nm. Figure 2a shows that as we increase *w*_e_, the second and third bands are shifted to higher wavelengths. The higher-order mode contribution (red part of the bands) is clearly more sensitive to *w*_e_ variations than the fundamental mode (blue part of the bands) since the higher-order mode is more localized inside the transverse elements of the periodic structure. Having control of the higher-order-mode cut-off frequency enables us to design for which wavelengths the slow light effect of this mode occurs, thus obtaining the desired bimodal behaviour. Figure 2b shows the maximum group velocity difference between both modes in the higher-order-mode slow light region and the bandwidth of the bimodal ROI as a function of *w*_e_. As previously explained, the group velocity difference in the ROI increases with *w*_e_ until we reach wavelengths near the PBG, where the fundamental mode also becomes slow light. The bandwidth decreases due to the flattening of the higher-order mode, resulting in smaller wavelength bimodal regions. We selected a *w*_e_ value of 1400 nm, as this configuration provides a high group velocity difference and a larger bandwidth. The critical effect of *w*_e_ can also be seen if we calculate the interference pattern of the spectrum as a sinusoidal function of the phase shift calculated from the bands in Fig. 2a and for a given length. In Fig. 2c, we can observe how *w*_e_ plays a crucial role in obtaining a large number of destructive and constructive peaks. With a *w*_e_ of 1300 nm, only two interference peaks are formed in the ROI, while up to eight peaks are observed when this parameter is increased to 1400 nm, as if it were an MZI with one of its arms drastically longer than the other. Moreover, the slow light influence of the higher-order mode on the interference pattern periodicity (see Fig. 2c) should be noted, where more grouped peaks are obtained for those wavelengths where the group velocity difference is maximized.

**Fig. 3:**
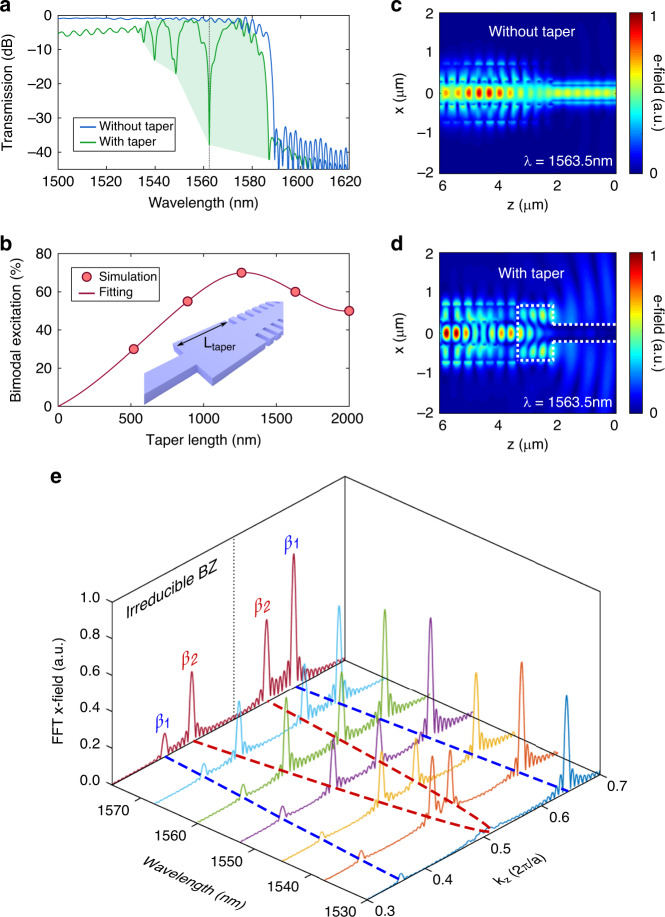
Simulation of the bimodal behaviour. **a** Transmission spectra for a bimodal PhC interferometer with and without a rectangular taper of 1200 nm with the previously detailed design parameters and for *N*=150 cell units repeated along the *z*-axis. The green shaded area represents the region of interest of the interferometer, corresponding to the bimodal region near the PBG. **b** Modal excitation obtained from the amplitude difference between constructive and destructive interference in the spectrum for different taper lengths. The inset details the value under consideration in a 3D sketch. **c**, **d** Electric field absolute value for the *xz* plane and *y* = 0 at the output interface between the bimodal periodic part and the single-mode waveguide with and without a rectangular taper between interfaces. **e** FFT amplitude of the electric field *x*-component along the propagation direction *z* for different wavelengths in the bimodal region. The dashed blue and red lines in the wavelength-*k*_*z*_ plane represent the dispersion relations of the fundamental and higher-order modes, respectively. The irreducible and second Brillouin zones are depicted

To study the interferometric behaviour of the device, the complete configuration with single-mode waveguides of 450 nm width as input and output ports was analysed. The transmission spectra for a length *N* = 150 elements are depicted in Fig. 3a, with and without a rectangular taper of 1200 nm between the end of the single-mode waveguide and the bimodal periodic waveguide. It can be observed that the response is nearly flat when the taper is not present, since only the fundamental mode is propagated through the 1D PhC. With a rectangular taper, the higher-order mode is properly excited, and thus, the interference pattern is clearly observed. Moreover, as was previously shown in Fig. 2c, the interference pattern is more grouped at lower wavelengths, demonstrating the dispersive behaviour of the higher-order mode in this region. This behaviour appears for wavelengths of ~1532 nm near the end of the irreducible Brillouin zone for the higher-order mode, where it becomes slow light. At even lower wavelengths (i.e., below 1530 nm), only the fundamental mode propagates, and thus, a mono-modal response of the transmission spectrum is observed. The bimodal excitation is optimized for a range of taper lengths in Fig. 3b, calculated as the difference between a maximum and a minimum peak caused by modal interference. An optimal amplitude modulation of ~70% for a taper of 1200 nm long is obtained for the modal interference nearest to the PBG. However, note that the bimodal excitation is decreased for lower wavelengths, as shown in the green shaded area of the ROI in Fig. 3a. To clarify the bimodal behaviour at the interfaces, the absolute value of the 2D-plane electric field at the output of the bimodal interferometer is depicted with and without a taper for destructive interference at 1563.5 nm in Fig. 3c and d, respectively. In the absence of a rectangular taper, all the energy is transferred to the single-mode output waveguide, while almost no power is transmitted to the output waveguide when the taper is present due to the proper bimodal excitation, which creates destructive interference.

To demonstrate that the fringes in the spectrum are due to modal interference, the 1D electric field *x*-component along the *z*-axis for *y* = 0 in a *N* = 150 bimodal periodic structure is calculated. By applying the Fast Fourier transform (FFT) over the propagating field, we obtain the wave vectors that are excited in the bimodal region. The results are shown in Fig. 3e as a function of the normalized wave vector axis and for different wavelengths. Each peak of the FFT corresponds to a propagating mode inside the periodic waveguide. As shown, the wave vectors of the modes inside the irreducible Brillouin zone are obtained, as well as those for the unfolded region between *k*_*z*_ = 0.5 2*π*/*a* and *k*_*z*_ = 0.7 2*π*/*a*. This second Brillouin zone is a mirror image of the first region and provides information about the modes and their dispersion characteristics. Therefore, perfect agreement is obtained between the positions of the peaks and the band diagrams previously calculated by using two different simulation methods, which enables us to clarify the modal excitation inside the 1D PhC. Note that the fundamental mode at *k*_*z*_ ∼ 0.65 2*π*/*a* has a higher intensity than the higher-order mode at *k*_*z*_ ∼ 0.45 2*π*/*a*, which means that both modes are not equally excited and explains the results obtained in Fig. 3a regarding the interference pattern amplitude in the transmission spectrum.

### Experimental demonstration of slow light bimodal behaviour

**Fig. 4:**
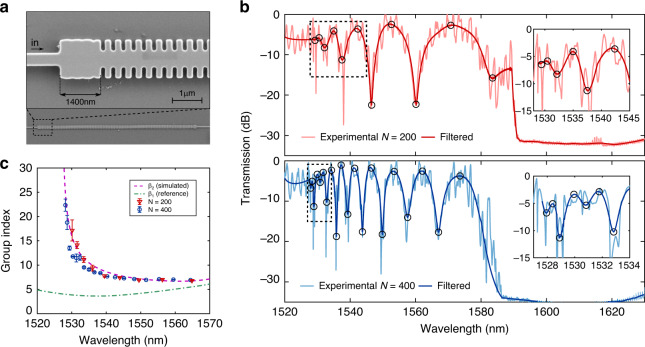
Experimental measurements of slow light bimodal behaviour. **a** Scanning electron microscopy (SEM) image of the fabricated silicon structures without a silica cover layer. A detailed picture of the interface between the single-mode and bimodal sections is shown with the presence of a fabricated rectangular taper of ~1400 nm in length. **b** Normalized experimental transmitted spectra for *N* = 200 and *N* = 400 periods under a silica cover (upper and lower graphs, respectively). The spectra were normalized with respect to a reference uniform waveguide measured under the same environmental conditions. Light colours represent the raw experimental data; dark colours refer to the filtered signal without the Fabry-Perot ripple created in the photonic circuit cavities. Constructive and destructive interferences are indicated by the black circles in both graphs. Insets depict a close-up view of the transmission spectrum in the slow light region. **c** Group index experimentally obtained from the maxima and minima interference points and with the simulated fundamental mode group index as a reference. Three different experimental measurements were carried out to obtain the error bars, depicted as the data standard deviation. The dashed line represents the theoretical results obtained from the band diagram simulations, which were shifted by only 5nm towards lower wavelengths to compensate for the small fabrication deviations

We fabricated bimodal interferometers with the design parameters previously detailed and a taper of nominal length (1200 nm) at the input–output interfaces (see Fig. 4a). All the parameters of the fabricated structures perfectly match the theoretical design except for the taper, which has a measured length of ~1400 nm. This difference occurs because the taper was very close to the first transversal element of the 1D PhC, with a gap of only 60 nm that was not resolved in the fabrication (see Fig. 4a). Despite this deviation, the fabricated taper length also remains within the optimal bimodal excitation range, as shown in Fig. 3b; thus, the experimental performance is not expected to be dramatically altered. Two different bimodal PhC lengths of 74 μm and 148 μm are considered, corresponding to numbers of periodic elements *N* = 200 and *N* = 400. The total length of the photonic sample is 0.9 mm, corresponding to the on-chip length between the input–output grating couplers. The experimental transmission spectra for both configurations are depicted in Fig. 4b. A PBG for wavelengths higher than 1590 nm can be observed, below which the bimodal ROI is obtained, as was theoretically predicted in the band diagram and propagation simulations. The raw experimental spectra, as well as the filtered data, are depicted to distinguish the bimodal interferences from the ripple caused by Fabry-Perot resonances. The free spectral range (FSR) of the ripple is homogeneously distributed and very similar to that calculated for an on-chip cavity distance of 0.9 mm (~1.5 nm), which demonstrates that this is caused by the Fabry-Perot contribution. Moreover, the results show the same FSR for the two different bimodal lengths considered; hence, the ripple must be caused by a common optical cavity in both designs (i.e., the resonances between the access grating couplers).

The positions of the maximum and minimum oscillations originating from the bimodal constructive and destructive interferences, which are marked with circles in Fig. 4b, are obtained by applying Lorentzian fitting over the filtered spectra. As expected, a higher number of bimodal interferences is observed in the spectrum for the *N* = 400 periods than for *N* = 200 as a result of the increment of the physical optical length of the interferometer. To evaluate the slow light behaviour of a PhC in standard MZI configurations^7^, the experimental group index of the active arm (*β*_2_ mode in our case) is calculated from the simulations of the reference arm (*β*_1_ mode). Therefore, the spectral dependence of the group index in the higher-order slow light mode can be deduced from the positions of the maximum *λ*_max_ and minimum *λ*_min_ spectral fringes as:

1 $$n_{\mathrm{g}}^{\beta 2}\left( {\lambda} \right) = \frac{{{\lambda }}_{{\mathrm{max}}}{\lambda}_{{\mathrm{min}}}}{{2L({\lambda}_{{\mathrm{max}}} - {\lambda}_{{\mathrm{min}}})}} + n_{\mathrm{g}}^{\beta 1}({\lambda})$$

where *L* is the length of the bimodal PhC waveguide and $n_{\mathrm{g}}^{\beta 1}$ is the group index of the fundamental mode acting as a reference signal. The red and blue markers in Fig. 4c represent the experimental group index in the *N* = 200 and *N* = 400 configurations by using the equation described above. The bimodal slow light interferometer exhibits a group index up to ~23 for the higher-order mode, which perfectly matches the simulations. These simulated values were calculated from the propagation constants of both modes considered in the bimodal band region, demonstrating that the oscillations marked with circles in Fig. 4a and b are due to the bimodal contribution. The simulated group index of the fundamental mode used in Eq. (1) is shown in green in Fig. 4c and presents lower group velocity values below 5 in the ROI.

### Interferometric performance in dynamic systems

Once the static response is provided, to calculate the phase shift accumulated by an induced change in the RI, we must also consider the spectral shift of a given interference peak. Moreover, due to the dispersive behaviour of our proposed structure, the FSR caused by the bimodal interferences varies along the ROI, yielding lower values in the slow light region. Hence, the mean value between two contiguous FSRs is used to obtain the experimental phase shift, which is calculated as follows:

2 $$\Delta \varphi ({\lambda}) = \frac{{2\Delta {\lambda}}}{{({\mathrm{FSR}}_{\mathrm{H}} + {\mathrm{FSR}}_{\mathrm{L}})}}$$

where Δ*λ* is the wavelength shift of the minima produced for the induced RI changes and FSR_H,L_ is the free spectral range at higher and lower wavelengths with respect to a given minimum interference peak, respectively.

**Fig. 5:**
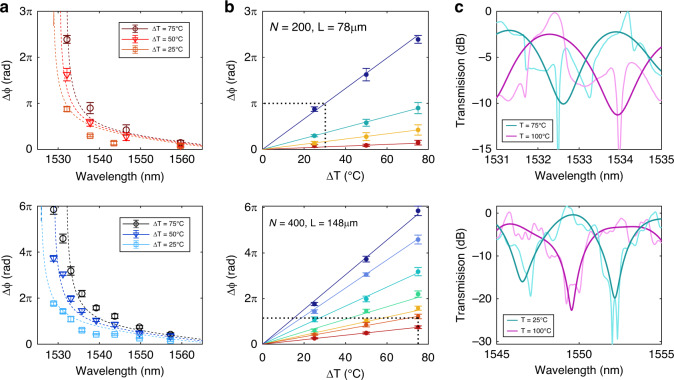
Optical response for changes in the silicon temperature. **a** Experimental phase shift obtained in the fabricated bimodal 1D PhC interferometer as a function of the wavelength for the different temperature increments considered and for the *N* = 200 and *N* = 400 configurations (upper and lower graphs, respectively). Dashed lines represent the band diagram simulation results, shifted 5 nm as in the previous case. **b** Evolution of the phase shift measured for a linear increment in the chip temperature. For the *N* = 200 configuration in the upper graph, different minima interference wavelengths at 1559.68 nm, 1546.51 nm, 1537.76 nm and 1532.13 nm are shown, with the increase in phase sensitivity represented by the shift from red to blue. For the *N* = 400 configuration, the minima interferences are placed at 1557.24 nm, 1549.73 nm, 1543.73 nm, 1539.23 nm, 1535.75 nm, 1533.08 nm, 1530.54 nm and 1528.95 nm and are represented by the lines transitioning from red to blue in the lower graph. Five different experimental measurements were carried out to obtain the error bars depicted. **c** The upper graph represents a close-up view of the normalized transmission spectrum for the *N* = 200 configuration (a bimodal 1D PhC length of 74 μm) working in the slow light region for a temperature change of 25 °C, corresponding to an accumulated phase shift of 0.9*π*. The lower graph represents a close-up view of the normalized transmission spectrum for the *N* = 400 configuration (a bimodal 1D PhC length of 148 μm) working in the normal light region for a temperature change of 75 °C and corresponding to an accumulated phase shift of *π*. For both graphs, raw and filtered data are represented by light and dark colours, respectively

To evaluate the response of the device as an optical modulator, a Peltier heater is used to change the chip operating temperature. Figure 5a shows the phase shift obtained as a function of the wavelength for different temperature increments in the *N* = 200 and *N* = 400 configurations. In both cases, the effect of slow light is clearly seen at wavelengths of ~1530 nm, where a drastic increase in the phase shift is obtained, which is also in good agreement with the simulations. Note that the *N* = 400 interferometer results are twice as high as those of the shorter case due to double the length being used. A fitting of the phase shift evolution is depicted in Fig. 5b for different destructive minima peaks when the temperature is varied, presenting perfectly linear behaviour for the available interferences. In the slow light region, phase shift values up to *π* for temperature increments of 30 °C are obtained at the 1532 nm interference, corresponding to a required silicon RI change of 5.4 × 10^−3^ for a *L*_*π*_ length of just 78 μm (see dashed line in the upper graph of Fig. 5b). Likewise, for the 148-μm-long interferometer at 1529 nm, 13.2 °C is required for a *π* phase shift, corresponding to a required silicon RI change of 2.4 × 10^−3^. By contrast, outside the slow light region, phase shift values of *π* are obtained for a temperature change of 75 °C at the 1550 nm interference in the 148-μm-long configuration, corresponding to a required silicon change in refractive index units (RIU) of 1.35 × 10^−2^ (see dashed line in the lower graph of Fig. 5b). Nevertheless, even in this regime, efficient interferometers with a large bandwidth are proved for optical modulation, which also demonstrates scalable design flexibility depending on the desired operation.

Examples of the measured spectra for the different temperatures considered are shown in Fig. 5c for slow light and normal operations. The filtered signal is also depicted to clearly reveal the modulating response in the transmitted spectra between the on and off states. In addition, it should be noted that the insertion losses and the extinction ratio of the raw data depend strongly on the ripple of the Fabry-Perot resonances. Nonetheless, if we consider the filtered spectra as the contribution of the bimodal behaviour, the insertion losses are ~2.5 dB for the upper graph in Fig. 5c and almost negligible in the case of the lower graph. In turn, an extinction ratio of ~10 dB is obtained in the slow light region, which increases up to ~20 dB outside this regime as a result of the improved bimodal excitation predicted theoretically.

**Fig. 6:**
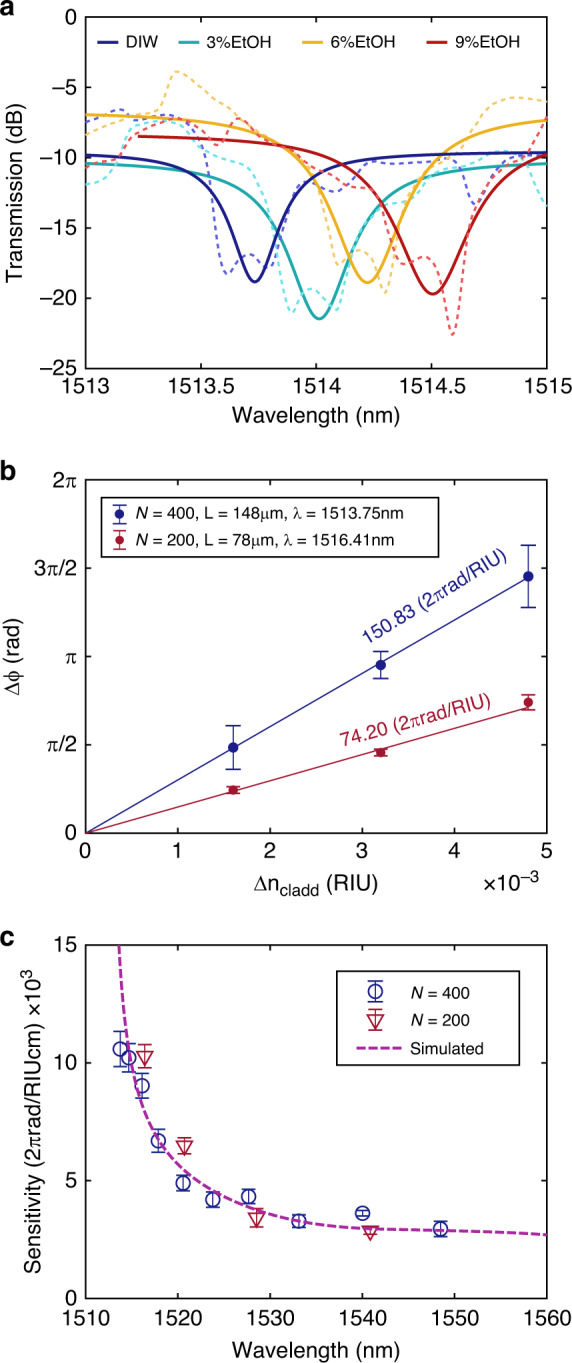
Optical response for changes in the cladding refractive index. **a** Normalized experimental spectra obtained for the *N* = 400 configuration fabricated without a silica cover layer when the structure is covered with different ethanol concentrations in DIW dilutions. Raw data are depicted as dashed lines, while the Lorentzian fitting over the filtered spectra, removing the Fabry-Perot contribution, is represented as solid lines. **b** Phase shift measurements for both the *N* = 200 and *N* = 400 configurations as a function of the linear increments in the cladding RI. **c** RI sensitivity experimentally obtained for *N* = 200 and *N* = 400 bimodal 1D PhC sensors as the slope of the linear fitting for different changes in the cladding RI. These values are scaled to a 1 cm length to compare our results with the literature. The pink dashed line represents the simulated sensitivity curve obtained from the theoretical band structures under similar conditions

Additional slow light bimodal structures without the silica upper cladding have also been fabricated to investigate the performance of the interferometer as a sensor. The design parameters are the same as those previously used to evaluate the operation as an optical modulator by means of temperature changes. Its transmission spectra when different ethanol dilutions in deionized water (DIW) are deposited over the 1D PhC interferometric structure are shown in Fig. 6a. Specifically, ethanol volumes in DIW of 3%, 6% and 9% are considered, corresponding to linear RI increments with respect to pure DIW of 1.6 × 10^−3^, 3.2 × 10^−3^ and 4.8 × 10^−3^ RIU according to the literature^52^. As can be observed, an interferometric response is now obtained for lower wavelengths (fringes located around 1515 nm are now tracked) since the DIW-based upper cladding has a lower RI than that of the silica. The spectrum is shifted towards higher wavelengths when increasing the RI of the cladding. As in the case of temperature changes, a Lorentzian fitting is processed over the spectral interferences to properly obtain the minima peaks caused by the bimodal behaviour (see Fig. 6a). Likewise, by knowing the spectral shift due to cladding RI changes, we can calculate its corresponding phase shift by using Eq. (2). Figure 6b shows the accumulated phase shift for the interferences with the highest sensitivity in the slow light region for a linear increment in the cladding RI for both the *N* = 200 and *N* = 400 configurations. The experimental sensitivity can be determined as the slope of the phase shift fitting for different cladding RI changes. Experimental values up to 75.20 2*π*rad/RIU and 150.83 2*π*rad/RIU are obtained for bimodal interferometer lengths of 74 μm and 148 μm, respectively. The obtained results clearly follow a linear evolution and present a twofold higher value due to the double length used for this configuration in comparison to the shorter one, similar to the previous case. In addition, the experimental results are compared to the simulations by applying the following equation to calculate the phase sensitivity of the interferometer:

3 $$S\left( {\lambda} \right) = \frac{{\Delta \varphi }}{{\Delta n_{\mathrm{c}}}} = \frac{{2\pi L}}{{\lambda }}\left( {\frac{{\partial n_{{\mathrm{eff2}}}}}{{\partial n_{\mathrm{c}}}} - \frac{{\partial n_{{\mathrm{eff1}}}}}{{\partial n_{\mathrm{c}}}}} \right)$$

where *n*_c_ is the cladding RI, *L* is the interferometer length and *n*_eff1,2_ is the effective index of the fundamental and higher-order modes, respectively. In standard MZI schemes in which one of the arms is completely isolated, the phase sensitivity is related only to the variation in the sensing arm effective index. In our case, both modes interact with the cladding variations; thus, the sensitivity depends on the effective index difference between them. Since the fundamental mode is strongly confined with a low group index and the higher-order mode presents a high dispersive behaviour, outstanding sensitivity values are obtained for the proposed bimodal 1D PhC waveguide. The wavelength dependence of the sensitivity is depicted in Fig. 6c; as shown, its value dramatically increases in the slow light region, and perfect agreement with the simulations is presented. To compare these results with those of other interferometers in the literature with different lengths, values normalized to 1 cm are also calculated, corresponding to almost identical experimental sensitivities up to 10.62 × 10^3^ 2*π*rad/RIU cm and 10.19 × 10^3^ 2*π*rad/RIU cm for the *N* = 200 and *N* = 400 configurations, since they are normalized to a common length. Likewise, outside the slow light region, values around 3.5 × 10^3^ 2*π*rad/RIU cm for a large operation range of 30 nm are experimentally demonstrated, which are not as good as those in the slow light region but still higher than those of other interferometric configurations. In addition, the spectral sensitivity in nanometres per RI unit is calculated, yielding values of 138.75 nm/RIU for those minima spectral dips occurring in the slow light regime shown in Fig. 6a. These values are in the range of the spectral sensitivities reported for similar slow light elements in the MZI configurations^45^. However, it should be noted that the spectral sensitivity may change outside the slow light regime due to the highly dispersive behaviour of the structure.

## Discussion

In conclusion, we have proposed and demonstrated the possibility of obtaining optical interferometers by using a single-channel bimodal periodic structure. Compared to other bimodal configurations^46–49^, our design makes use of 1D PhC structures with an active mode working in the slow light regime, which is translated into a significantly higher phase-shift sensitivity to induced RI changes. This effect has been realized by engineering the periodic unit cell to achieve active control of the band structure to optimize the desired bimodal behaviour. An experimental group index of 23 was measured for the higher-order mode supported by the fabricated 1D PhC. This value is in the range of those results reported in other works in which similar 1D periodic structures were included in an MZI^44^ but not as high as for works where 2D PhC structures were directly used to create an MZI^7^. This last result encourages us to look for other PhC configurations in which the single-channel bimodal slow wave behaviour can be further exploited to reduce the interferometer footprint and further improve the performance. Further optimization of the spectral response must be addressed in future experimental work for the current design, especially to reduce the Fabry-Perot ripple and improve the modulation depth and losses in the slow light regime.

The proposed device has also been experimentally validated for optical modulation and sensing purposes to determine its efficiency in dynamic systems. By changing the temperature of the chip, we can test the response of the interferometer to small changes in the RI of the silicon structure. In comparison with other interferometers that include slow light elements^38,39^, we propose a highly efficient temperature modulation where a change of only 30 °C is required to achieve phase shifts of *π* in a single-channel interferometer with a footprint of only ~100 μm^2^. This size means a reduction of two orders of magnitude with respect to conventional MZI structures^28^ and of more than one order of magnitude with respect to compact MZI modulators^29^ and other interferometric schemes, including those based on the use of 1D and 2D PhCs^44,53^. Due to its extremely reduced design, this type of bimodal PhC silicon waveguide may be used for the integration of multiple on-chip modulators^54^, as well as for the implementation of matrix multiplications of several input binary signals for making all-optical programmable logic devices. However, switching speed limitations as a result of using thermo-optic effects^43^ must be addressed in further developments. The sensing operation has also been demonstrated for different ethanol dilutions in DIW, corresponding to a linear change in the RI of the cladding. Experimental sensitivities of 10^4^ 2*π*rad/RIU cm are reported, which indicate an improvement by a factor of more than 10 with respect to the traditional MZI configurations^30^ and of ~7.5 for slot-based MZIs and silicon nitride bimodal waveguides^46,55^. These sensitivity results are similar to those obtained for other slow light MZI sensors^45^ but in our case integrated in a significantly more compact single-channel structure. Furthermore, its straightforward monolithically formed design in silicon offers remarkable advantages for mass integration and low-cost production with significant implications for optical network interconnects or lab-on-a-chip instruments, among others.

## Materials and methods

Numerical simulations to obtain the band diagrams of the basic 1D PhC unit cell were carried out using the MIT Photonics Band (MPB) free software, which computes definite-frequency eigenstates of Maxwell’s equations in periodic dielectric structures. It employs the plane wave expansion (PWE) numerical method in fully vectorial and 3D spaces. In particular, we used silicon (*n* = 3.477) for the periodic structure, with a thermo-optic coefficient of 1.8 × 10^−4^/K, and silica (*n* = 1.444) for the substrate and cladding, with a grid step of 10 nm, as the mesh. The first five TE-like bands were computed, including the first three that present an even parity with respect to the *x* = 0 plane. The simulations of the transmission spectra and the field excitation were numerically calculated using the software package CST Microwave Studio. In more detail, we used a fully vectorial 3D time domain solver using finite-integration techniques to simulate the whole interferometric system, including the single-mode and bimodal waveguides. A hexahedral grid of 20 cells per wavelength was used for the entire structure, with silica as the background. In turn, the FFT of the field along the *z*-axis was obtained using 5000 points to obtain the propagating modes in the bimodal section. The excitation of the structure was provided by using standard waveguide ports at the input and output to provide the scattering parameters.

The photonic structures were fabricated on a silicon-on-insulator (SOI) wafer with a silicon layer thickness of 220 nm and a silica buried layer of 2 µm. An acceleration voltage of 30 KeV and an aperture size of 30 µm were used in an electron-beam lithography process to create the photonic structures on a hydrogen silsesquioxane (HSQ) negative resist, and then inductively coupled plasma etching was used to transfer the designed patterns onto the silicon layer. For the experimental characterization, a continuous wave (CW) tuneable laser (Keysight 81980) and a coherent TE polarizer were used to vertically couple the light into photonic structures by using cleaved optical fibres close to the grating couplers. At the output, a power metre (Keysight 81636B) synchronized with the laser measured the response of the optical circuits. The transmitted spectra were digitally recorded using a LabVIEW application, which was also responsible for controlling the chip temperature by using a Peltier heater connected to the copper holder of the photonic sample. A time frame of 5 min was used after the temperature changes to let the sample stabilize at the desired conditions. For the sensing experiments, the chip was placed on an airtight container and covered with increasing ethanol in DIW dilutions directly dropped onto the sample and measured after a 5-min stabilization period.
